# Early postnatal C-reactive protein elevation during initial hospitalization in neonates with giant omphalocele undergoing delayed repair

**DOI:** 10.3389/fped.2026.1743292

**Published:** 2026-03-05

**Authors:** Zilu Huang, Yanfen Peng, Junjian Lv, Wei Zhong, Qiuming He

**Affiliations:** Department of Neonatal Surgery, Guangzhou Women and Children’s Medical Center, Guangzhou Medical University, Guangzhou, China

**Keywords:** delayed surgery, omphalocele, peak CRP, primary surgery, sterile inflammation

## Abstract

**Introduction:**

To preliminarily describe the dynamic changes and clinical characteristics of serum C-reactive protein (CRP) in giant omphalocele (GO) neonates with delayed repair during the early postnatal period.

**Methods:**

A retrospective study included 15 neonates with GO who underwent delayed repair at our hospital. CRP was collected at 0, 5, 7, 10, 14, 21 days after birth and before discharge. Data on hospital stay duration, complications, and anti-infection treatment were recorded. Descriptive statistics were used to present the trend of CRP changes, and the relationship with clinical indicators was preliminarily analyzed.

**Results:**

Among the 15 full-term neonates (average gestational age 38.5 weeks, birth weight 2,821 g), CRP reached a peak value on the 5th day after birth (median 87.1 mg/L), followed by a decreasing trend but remaining at a high level (78.0, 67.2, and 48.0 mg/L on the 7th, 10th, and 14th days, respectively). The peak CRP level was positively correlated with the hospital stay duration (R = 0.78, *p* = 0.001). 73.3% (11/15) of the GO neonates received empirical antibiotic treatment (average course of 11 days), while the pathogen positivity rate was only 20% (3/15), and all were cultured from the sac membrane secretions. Based on the duration of continuous CRP elevation as the classification criterion, it was found that neonates with a longer duration of elevated CRP had larger defects, longer hospital stays, and longer time to achieve full enteral nutrition.

**Conclusion:**

Neonates with GO undergoing delayed repair exhibit significant CRP elevation in the early postnatal period, but this rise correlates poorly with proven infection. Clinicians need to carefully interpret the changes in CRP and avoid excessive anti-infection treatment. This retrospective study provides preliminary data and hypothesis basis for subsequent large-sample studies.

## Introduction

Omphalocele is the most common congenital abdominal wall defect in newborns, occurring in approximately 2.6 per 10,000 live births (1). Cases with a defect diameter of ≥5 cm or with the liver protruding outside the body cavity are classified as giant omphalocele (GO), accounting for 10%–42% of all cases (2). With the widespread implementation of prenatal screening and the advancements in perinatal treatment, the survival rate of neonates with GO has risen to 70%–90%. However, the incidence of combined malformations, pulmonary dysplasia, and prolonged hospital stays remains markedly higher in GO neonates than that of normal newborns (1, 2).

Currently, the repair strategies for GO are categorized into primary repair, staged silo reduction with delayed fascial closure and delayed repair. Among these, delayed repair, achieved through non-operative conservative management using topical escharotics to promote epithelialization without an attempt of fascial closure, can minimize the risk of abdominal compartment syndrome to the greatest extent and has been adopted by numerous pediatric surgical centers. Nevertheless, the delayed repair approach introduces additional challenges. During the early postnatal period, neonates experience a loss of the peritoneal barrier, and the presence of extensive fibrin eschar with exposed underlying intestines creates potential sites for infection. Despite this risk, there are currently no reliable early markers for infection in clinical settings.

C-reactive protein (CRP), owing to its ease of measurement, is commonly used in neonatal intensive care units to screen for bacterial infections and to guide the duration of antibiotic therapy. It has been generally accepted that a single CRP cut-off of ≥10 mg/L predicts culture-proven early-onset neonatal sepsis. However, in the group of GO neonates undergoing delayed repair, the typical range of CRP fluctuations has not yet been established. Previous studies suggest that even in GO infants without clinical signs of infection, CRP levels can rise to 50–70 mg/L within several days after birth, leading to unnecessary prolongation of antibiotic therapy, extended intravenous nutrition, and increased parental anxiety (3, 4). Applying the CRP threshold for normal values in newborns without critical evaluation may obscure genuine infections or lead to unnecessary treatment. Unfortunately, most existing literature focuses on survival rates, respiratory outcomes, or long-term follow-up in gestational offspring, leaving a critical knowledge gap concerning the dynamic changes in CRP during the early postnatal period. Only one study briefly mentioned a prolonged inflammatory state in GO neonates managed with delayed repair, which was associated with pulmonary hypertension but lacked definitive evidence of infection (4). Consequently, clinicians must rely on empirical judgment to determine whether elevated CRP levels indicate a physiological tissue injury response or a potential infection, and whether antibiotic therapy should be discontinued in the GO population. This knowledge gap has directly hindered the standardization and widespread adoption of the delayed repair approach.

Based on the background above, we hypothesize that in the early postnatal period, newborns with GO who underwent delayed repair generally exhibit elevated CRP levels, and the extent of this elevation is related to clinical complications but does not necessarily indicate infection. This single-center retrospective cohort study systematically described the dynamic CRP curves of 15 neonates with GO who underwent delayed repair during their initial hospitalization. It preliminarily summarized their clinical characteristics and the association with short-term outcomes, providing baseline data for the subsequent development of the CRP interpretation criteria and antibiotic discontinuation guidelines for this specific population.

## Materials and methods

A single-center, retrospective observational study was conducted in the surgical neonatal intensive care unit of our center. The study was approved by Guangzhou Women and Children's Medical Center Ethics Committee, with a waiver of informed consent due to its retrospective nature. Neonates with GO admitted between January 2016 and July 2025 were screened. The neonates included in the study must meet all of the following criteria: 1) a defect size of ≥5 cm or the presence of liver in the sac; 2) initial management by conservative epithelialization without an attempt of fascial closure during the first admission; and 3) availability of at least three serum CRP measurements during the first admission. Neonates with GO were excluded if they had major chromosomal anomalies, a ruptured sac at birth, or fewer than three CRP measurements during the first hospitalization. Meanwhile, neonates undergoing primary omphalocele repair who were admitted to our hospital during the same period served as the control group. Baseline variables were obtained from electronic medical records and included gestational age (GA), birth weight (BW), Apgar scores, defect diameter, presence of liver herniation, and associated anomalies. The dynamic analysis of CRP primarily encompassed measurements on days (D) 0, 5, 7, 10, 14, or then followed by weekly assessments until discharge. Parameters analyzed included the peak CRP value and the number of consecutive days with CRP ≥20 mg/L. Consistent with the work of Teillet B et al. (4), we defined this CRP threshold as the optimal discriminator for chronic inflammatory states derived from a similar study population comparable to ours.

Additionally, the duration of total parenteral nutrition (TPN), length of initial hospital stays, time to achieve full enteral nutrition, major complications, and antibiotic courses were evaluated. Here, the main complications were primarily infection-related, focusing on sepsis and local sac infections. Sepsis was defined as positive blood culture and/or presence of ≥2 systemic inflammatory response syndrome criteria with suspected infection, classified by onset timing as early-onset (≤72 h) or late-onset (>72 h). Local sac infection was defined as positive cultures of sac membrane secretions in the absence of positive blood cultures. Blood cultures were obtained immediately after birth, and sac membrane cultures were performed prior to wrapping. Both were rechecked when neonates showed clinical signs of suspected infection (e.g., elevated CRP or fever). Blood cultures were performed using an automated continuously monitored system, with > 1 ml of blood inoculated into pediatric aerobic bottles. Cultures were incubated for 7 days or until positivity.

Normality was assessed by the Shapiro–Wilk test. Continuous data are presented as median (interquartile range, IQR) and compared using the Mann–Whitney *U*-test. Categorical variables are reported as *n* (%) and analyzed with Fisher's exact test. The correlation between peak CRP and hospital stay was evaluated by Spearman's *ρ*. A two-tailed *p*-value of <0.05 was considered statistically significant. All analyses were performed with SPSS version 26.0.

## Results

### Inclusion process and baseline characteristics

From January 2016 to July 2025, a total of 20 neonates with GO who underwent delayed repair were admitted to our center. According to the inclusion and exclusion criteria, 15 neonates were ultimately included in the retrospective analysis. The median defect diameter was 8.0 cm (IQR, 6.0–10.0 cm), and all cases involved liver herniation. Three neonates (20.0%) had congenital heart malformation, respectively ventricular septal defect, atrial septal defect, and atrial septal aneurysm (Table 1). All GO newborns received local bandaging during their initial hospitalization to promote epithelialization and did not undergo any surgical intervention.

**Table 1:** Clinical characteristics of neonates with omphalocele treated with delayed repair or primary surgery.

Variables	GO with delayed repair (*n* = 15)	Omphalocele with primary surgery (*n* = 19)	*p*-value
Gestational age (weeks, IQR)	38.6 (38.0–39.0)	38.0 (37.3–38.6)	0.088
Birth weight (g, IQR)	2,840 (2,560–3,040)	3,200 (2,825–3,560)	0.016
Male sex (*n*, %)	6 (40.0%)	12 (70.6%)	0.153
Apgar score at 5 min (IQR)	10 (9–10)	10 (9–10)	0.518
Defect diameter (cm, IQR)	8.0 (6.0–10.0)	4.0 (3.3–5.0)	<0.001
Liver herniation (*n*, %)	15 (100%)	6 (35.3%)	<0.001
Congenital heart malformations (*n*, %)	3 (20%)	0	0.092
Late-onset sepsis (n, %)	1 (6.7%)	0	0.469

### Dynamic CRP curve and peak characteristics

**Figure 1:**
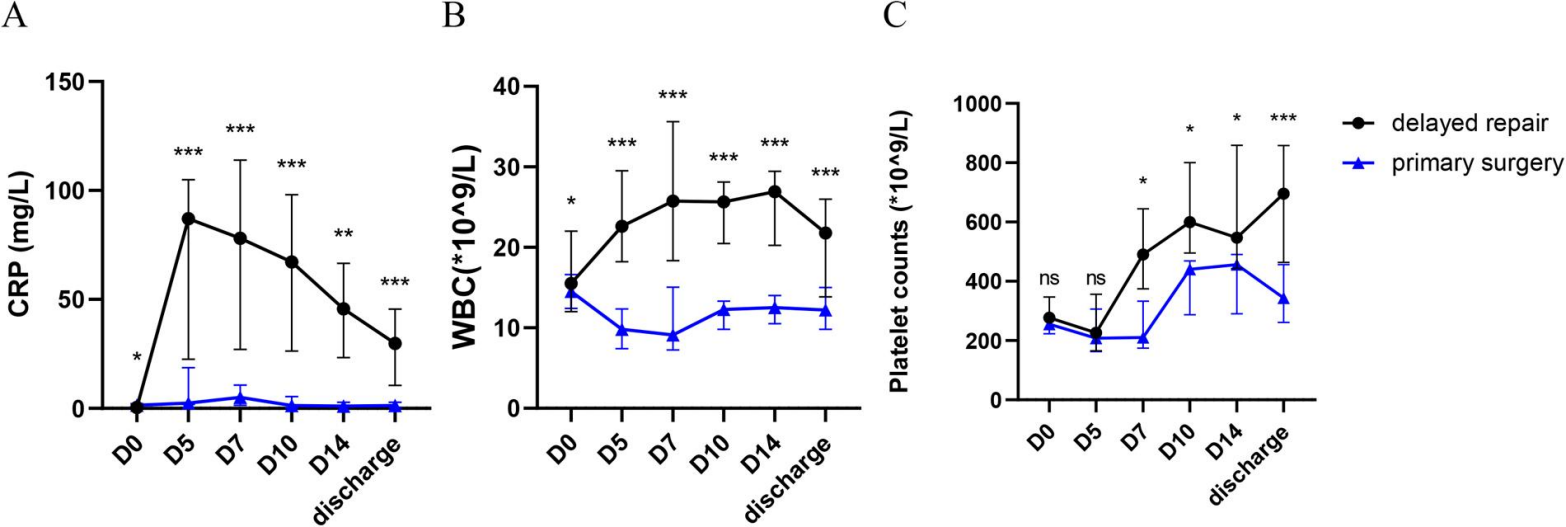
Comparison of inflammatory markers in neonates with omphalocele undergoing delayed repair vs. primary surgery. Serum C-reactive protein (CRP) **(A)**, white blood cell count (WBC) **(B)**, and platelet count (PLT) **(C)** in neonates with omphalocele undergoing delayed repair (*n* = 15) vs. primary surgery (*n* = 19). Data were collected at postnatal days 0, 5, 7, 10, 14 and before discharge. Box plots show median with interquartile range (IQR). ns: not significant, *p < 0.05, **p < 0.01, ***p < 0.001 vs. primary surgery group at the same time point.

**Figure 2:**
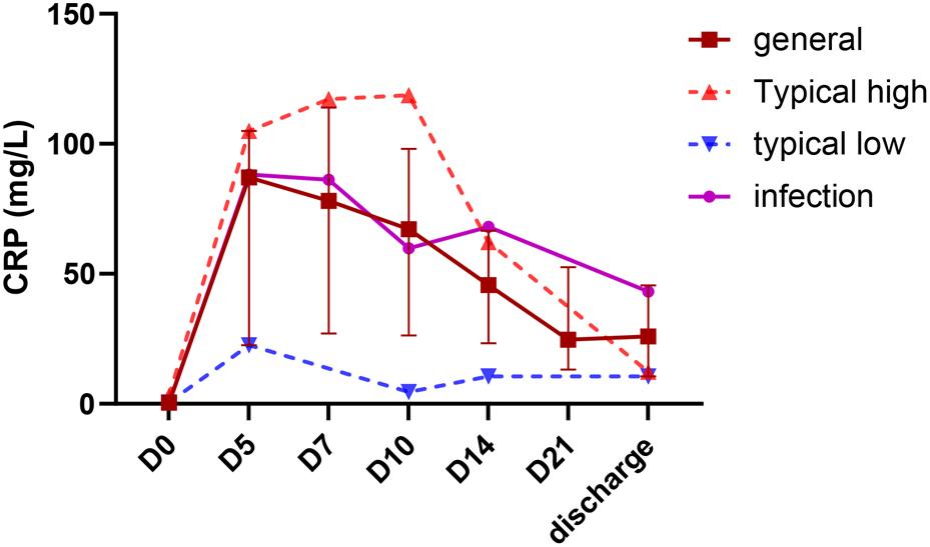
Representative CRP trajectories in neonates with giant omphalocele. The brown line represents the cohort median at each time point (postnatal day 0, 5, 7, 10, 14, 21, and before discharge). Three representative individual trajectories illustrate different inflammatory patterns: orange line—patient with persistently elevated CRP (peak >100 mg/L) despite negative cultures (typical high); blue line—patient with consistently low CRP throughout hospitalization (typical low); purple line—patient with confirmed positive culture of sac membrane (infection).

Fifteen neonates exhibited elevated CRP levels, peaking on day 5 (D5) after birth with a median value of 87.1 mg/L, followed by a decreasing trend but remaining at a high level (78.0 mg/L, 67.2 mg/L, and 48.0 mg/L on the D7, D10, and D14, respectively). Except for D0, CRP values in the GO neonates who underwent delayed repair were significantly higher at subsequent time points compared to those who received the primary surgery (Figure 1). Notably, the two groups showed no significant differences in baseline clinical characteristics, including GA, sex, and APGAR scores (Table 1). The peak value of CRP in GO neonates with delayed repair was also significantly greater than that of 19 neonates with omphalocele who underwent primary surgical repair during the same period [103.9 (56.1–148.8) vs. 2.9 (1.5–8.5) mg/L, *p* < 0.001]. Individual CRP trajectories were heterogeneous. To avoid visual clutter, only the median trend with IQR shading and three representative curves (low, high, and infected) are displayed in Figure 2.

### Infection outcomes and blood culture results

Five neonates presented with fever, two of whom had positive cultures from the sac membrane secretions (one case of Staphylococcus haemolyticus on D5 and one case of Bacillus cereus on D10). All blood cultures from the GO newborns were negative. Additionally, one neonate with a local sac membrane infection had no fever, but the culture of the sac membrane secretion was positive for Enterobacter cloacae and Klebsiella pneumoniae on D4. Moreover, one neonate was diagnosed with late-onset clinical sepsis, presenting with bloody stool, cyanosis, and combined with pulmonary hypertension requiring inhaled nitric oxide treatment on D5. This case was accompanied by a significant increase in CRP and a marked decrease in platelet count, although the blood culture was also negative.

### Peak CRP level and duration of CRP elevation and clinical prognosis

**Figure 3:**
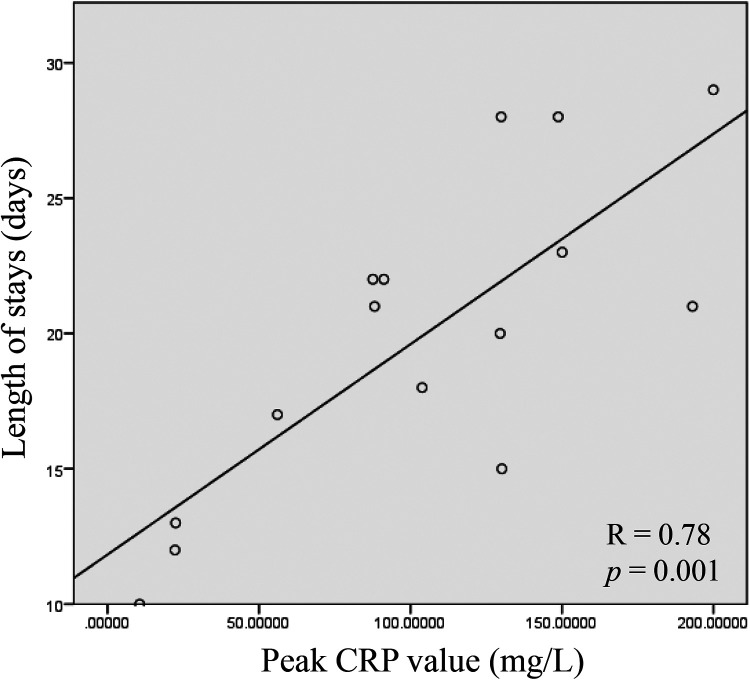
Correlation between peak CRP level and length of hospital stay in neonates with giant omphalocele. Scatter plot showing the relationship between peak CRP level (mg/L) during hospitalization and total length of stay (days) in neonates with giant omphalocele (*n* = 15). The solid line represents the linear regression fit. Spearman correlation coefficient R = 0.78, *p* = 0.001.

Spearman analysis showed that the peak CRP level was positively correlated with the length of hospital stay (R = 0.78, *p* = 0.001; Figure 3). Based on the threshold definitions of CRP for chronic inflammatory states from previous studies (4), we established the criterion that CRP levels exceeding 20 mg/L and remaining elevated for more than 15 days would serve as the grouping standard. Based on this, we divided 15 neonates with GO into two groups. The results indicated that neonates with prolonged CRP elevation duration had larger defect diameters, higher peak CRP levels, longer times to reach full enteral nutrition, and extended hospital stays (Table 2).

**Table 2:** Comparison of clinical outcomes subgrouped by duration of persistently elevated CRP.

Variable	Overall (*n* = 15)	CRP >20 mg/L ≥ 15 days (*n* = 9)	CRP >20 mg/L < 15 days (*n* = 6)	*p*-value
Defect diameter (cm, IQR)	8.0 (6.0–10.0)	10.0 (8.5–12.5)	6.0 (5.8–7.3)	0.003
Peak CRP (mg/L, IQR)	103.9 (56.1–148.8)	130.2 (96.0–171.6)	55.1 (19.4–100.8)	0.013
Composite complication^a^ (*n*, %)	4 (26.7%)	3 (33.3%)	1 (16.7%)	0.604
Feeding intolerance (*n*, %)	3 (20.0%)	3 (33.3%)	0	0.229
TPN duration (days, IQR)	2.0 (1.0–4.0)	2.0 (1.0–7.5)	2.0 (1.0–2.0)	0.321
Time to full enteral nutrition (days, IQR)	7.0 (4.0–14.0)	14.0 (7.0–18.5)	4.0 (3.0–6.3)	0.021
Hospital stays (days, IQR)	21.0 (15.0–23.0)	21.0 (17.5–28.0)	16.5 (11.5–22.0)	0.098

^a^ Composite complications included clinical sepsis and sac infections.

### Changes in other inflammatory indicators

Additionally, the white blood cell (WBC) counts consistently exceeded 20 × 10^9^/L starting from D5, which was significantly higher than that of the 19 neonates who underwent primary surgery during the same period. Platelet counts were also significantly higher on D7 compared to the controls, and the differences between the groups were statistically significant, as illustrated in Figure 1.

### Treatment outcome

Among the 15 neonates, 11 (73.3%) of them received antibiotic treatment, with a median starting time of 5.5 days after birth and an average duration of 11 days. The primary antibiotics used were third-generation cephalosporins. All neonates were clinically cured (stable vital signs, hemodynamic stability, feeding tolerance) and discharged from the hospital, with no cases of necrotizing enterocolitis or in-hospital mortality reported in the group. The median hospital stay was 21 days (IQR, 15–23 days), and no infection recurrence was observed during the 30-day follow-up.

## Discussion

Among 15 GO newborns undergoing delayed repair, CRP peaked at a median of 103.9 mg/L on around five days after birth, coinciding with epithelial scab formation from our previous study (5), and correlated with the length of hospital stay (R = 0.78, *p* = 0.001), despite no definitive evidence of infection. These findings suggest a physiological association with tissue damage and sterile inflammation, indicating that standard neonatal CRP thresholds may mislead management in this population and thereby supporting individualized interpretation.

Previous studies on GO have primarily focused on survival rates, respiratory outcomes, and long-term follow-up. A systematic review has shown that the mortality rate for neonates with GO treated with primary staged closure can be as high as 30%, with the main causes of death including pulmonary dysplasia, postoperative infection, sepsis, and abdominal compartment syndrome (6). Several single-center retrospective studies in China have analyzed the adverse prognosis of neonates with omphalocele. The results indicated that the postoperative infection rate among GO neonates was as high as 32%, with CRP levels exceeding 10 mg/L serving as an early predictor of infection (7). In a retrospective cohort study involving 1,240 GO neonates, systemic inflammation, characterized by elevated CRP (>20 mg/L) and WBC (>15 G/L), was identified as an independent risk factor for pulmonary hypertension. This condition significantly prolonged hospital stays and increased mortality (4).

However, in another study involving newborns with abdominal wall defects such as omphalocele and gastroschisis, it was found that CRP levels generally increased during the early postnatal period. Therefore, elevated CRP levels at the initial measurement or within the first 48 h did not provide sufficient discriminatory power for diagnosing sepsis or predicting adverse outcomes (3). In a multicenter retrospective cohort study conducted in the United States, it was found that the antibiotic usage rate for newborns with abdominal wall defects was as high as 93%, while the incidence of early-onset sepsis was only 1% (8). In this study, the infection rate was the highest among infants with GO (15.1%), while it was the lowest among infants with gastroschisis (5.5%) (8). The incidence of late-onset sepsis was higher in our delayed repair cohort (1/15, 6.7%) than in infants undergoing primary surgery (0/19), likely reflecting the increased infection risk associated with prolonged exposed sac membrane and delayed abdominal closure. However, this comparison is limited by our small sample size.

In summary, previous studies have extensively discussed the association between GO newborns and infections; however, specific data on CRP levels have not been clearly reported. CRP has generally been mentioned as a secondary indicator of infection, and its measurement is not standardized in the GO population. Furthermore, many medical centers have previously employed primary suture or silo bag staged reduction techniques, resulting in less tissue damage and consequently lower CRP levels. In contrast, due to limited resources and low economic affordability among parents in China, delayed repair has gradually become the mainstream treatment method for GO in recent years, which has amplified and highlighted the elevated CRP phenomenon.

When the newborns with GO experience delayed repair and lose the amniotic-peritoneal barrier, the surface of the exposed intestinal tube rapidly develops a fibrinous scab, which is comparable to a second-degree burn on the skin. This study observed that the increase in CRP was not significantly correlated with positive cultures, supporting the hypothesis of sterile inflammation. Unlike infection driven inflammation, sterile inflammation is triggered by cell necrosis and release of damage-associated molecular patterns (DAMPs), such as IL-1α and HMGB1 (9), which activate macrophages to produce pro-inflammatory cytokines (TNF-α and IL-6) and hepatic CRP synthesis, without requiring the involvement of lipopolysaccharide or bacterial DNA (10). The mechanism of sterile inflammation is well-described in trauma and atherosclerosis (9) but remains underexplored in omphalocele.

In neonates with GO, extensive loss of the epithelial barrier after birth causes epithelial necrosis due to traction, drying, or temperature fluctuations, generating DAMPs that may trigger sterile inflammation. A previous retrospective study has associated this prolonged inflammatory state with liver herniation, delayed abdominal closure, and pulmonary hypertension (4) while our study could not confirm the latter association due to limited sample size (only one infant exhibited pulmonary hypertension). Future studies with larger sample sizes will be necessary to better investigate the association between sterile inflammation and the short-term or long-term prognosis of GO neonates undergoing delayed repair. In the later stages of research, simultaneous measurement of procalcitonin (PCT), HMGB1/IL-1α, IL-6, and CRP levels would provide stronger evidence confirming this mechanism.

This study also has some limitations. First, the sample size was limited to only 15 cases, which precluded calculation of the optimal CRP cutoff value. Future studies with a sample size of at least 100 cases are needed. Second, PCT data were missing in 53.3% of neonates, precluding us from incorporating this more specific bacterial infection marker into our primary analysis. Also, novel inflammatory markers such as IL-6 and HMGB1 could be introduced to provide a more comprehensive assessment of systemic inflammation. Similarly, infection diagnosis currently relies on blood cultures, which are prone to false negatives. Combining this approach with 16S rRNA metagenomic sequencing in future studies may improve pathogen detection and standardize antibiotic use. Given that all infections occurred >72 h after birth in our cohort, cefoperazone-sulbactam (80 mg/kg IV q12 h) was chosen as first-line empiric therapy for its broad Gram-negative coverage, while meropenem (40 mg/kg IV q8 h) was reserved for one neonate with severe clinical sepsis. We recognize the lack of standardized protocols as a limitation, and future studies should prospectively evaluate optimal empiric regimens for this population. Finally, since delayed repairs of GO typically occur around one year after birth, long-term and complete follow-up was not feasible for all cases. Therefore, it remains unclear whether early elevated CRP levels affect long-term cognitive outcomes. A prospective randomized controlled trial investigating a “CRP-guided antibiotic discontinuation point” could help develop a more optimized treatment strategy for GO infants undergoing delayed repair.

In conclusion, elevated CRP levels in the early postnatal period of GO neonates with delayed repair appear to be associated with sterile inflammatory responses and the extent of scab formation, rather than bacterial infection. Both the peak CRP value and the duration of its continuous elevation correlate with the defect diameter, the length of hospital stay, and time to achieve full enteral nutrition. Therefore, further research is warranted to determine whether targeted inflammation management can improve the prognosis and treatment of both short-term and long-term complications.

## Data Availability

The datasets presented in this article are not readily available because the datasets are not publicly available due to hospital policy but may be shared in anonymized form upon reasonable request to the corresponding author and with approval of the institutional ethics committee. Requests to access the datasets should be directed to Qiuming He, qiuminghe@foxmail.com.
